# Comparison of Verona Integron-Borne Metallo-β-Lactamase (VIM) Variants Reveals Differences in Stability and Inhibition Profiles

**DOI:** 10.1128/AAC.01768-15

**Published:** 2016-02-26

**Authors:** Anne Makena, Azer Ö. Düzgün, Jürgen Brem, Michael A. McDonough, Anna M. Rydzik, Martine I. Abboud, Ayşegül Saral, Ayşegül Ç. Çiçek, Cemal Sandalli, Christopher J. Schofield

**Affiliations:** a Chemistry Research Laboratory, Department of Chemistry, University of Oxford, Oxford, United Kingdom; b Department of Genetics and Bioengineering, Gümüşhane University, Gümüşhane, Turkey; c Department of Biology, Coruh University, Artvin, Turkey; d Department of Medical Microbiology, Recep Tayyip Erdoğan University, Rize, Turkey; e Department of Biology, Recep Tayyip Erdoğan University, Rize, Turkey

## Abstract

Metallo-β-lactamases (MBLs) are of increasing clinical significance; the development of clinically useful MBL inhibitors is challenged by the rapid evolution of variant MBLs. The Verona integron-borne metallo-β-lactamase (VIM) enzymes are among the most widely distributed MBLs, with >40 VIM variants having been reported. We report on the crystallographic analysis of VIM-5 and comparison of biochemical and biophysical properties of VIM-1, VIM-2, VIM-4, VIM-5, and VIM-38. Recombinant VIM variants were produced and purified, and their secondary structure and thermal stabilities were investigated by circular dichroism analyses. Steady-state kinetic analyses with a representative panel of β-lactam substrates were carried out to compare the catalytic efficiencies of the VIM variants. Furthermore, a set of metalloenzyme inhibitors were screened to compare their effects on the different VIM variants. The results reveal only small variations in the kinetic parameters of the VIM variants but substantial differences in their thermal stabilities and inhibition profiles. Overall, these results support the proposal that protein stability may be a factor in MBL evolution and highlight the importance of screening MBL variants during inhibitor development programs.

## INTRODUCTION

**FIG 1:**
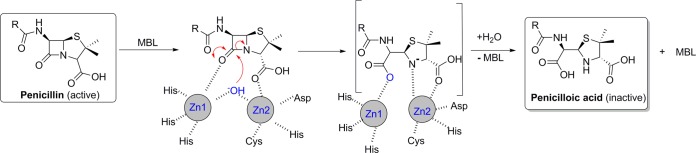
Outline scheme for MBL-mediated β-lactam hydrolysis.

Antibiotic resistance is a formidable threat to society; each year, an estimated 2 million people are infected with antibiotic-resistant bacteria in the United States alone, resulting in ∼23,000 deaths (1). β-Lactam antibiotics remain of immense clinical importance in the treatment of bacterial infections, but resistance increasingly compromises their clinical use. The most important mechanism of resistance to β-lactam antibiotics is mediated by β-lactamases, which catalyze β-lactam hydrolysis, thus inactivating the antibiotics (Fig. 1). β-Lactamases are broadly divided into four classes: those in classes A, C, and D employ a hydrolysis mechanism involving a nucleophilic serine residue, while class B comprises the metallo-β-lactamases (MBLs), which employ metal ions in catalysis (2). Although, to date, the serine enzymes have been the most clinically relevant β-lactamases, MBLs are of increasing clinical concern, in part due to their broad-spectrum activities. MBLs catalyze the hydrolysis of virtually all classes of β-lactams, with monobactams being the exception (3, 4). Acquired MBLs, encoded by DNA on mobile elements, have been reported in multiple major Gram-negative pathogens, including members of the Enterobacteriaceae and Pseudomonas and Acinetobacter species, making MBL-producing microorganisms a serious public health concern (5–7). MBLs are divided into the B1, B2, and B3 subgroups, with subgroup B1 MBLs having the most clinical relevance. Recent years have seen the worldwide spread of acquired subgroup B1 MBLs, most importantly the imipenemase (IMP), Verona integron-borne metallo-β-lactamase (VIM), and New Delhi metallo-β-lactamase (NDM) groups (8). Despite relatively low amino acid sequence identity (9, 10), all identified MBLs share an αβ/βα sandwich fold as well as a conserved active site, which binds two or, less commonly, one zinc(II) ion. The roles of the zinc(II) ions in catalysis include substrate binding, activation of “hydrolytic” water, and stabilization of reaction intermediates. The active sites of the MBLs are characterized by mobile loops, including a hydrophobic L3 loop (residues 60 to 67, according to the standard class B β-lactamase (BBL) numbering scheme [10]) and a hydrophilic L10 loop (residues 223 to 242), both of which are involved in substrate binding (8, 11–13).

The VIM enzymes currently constitute one of the largest groups of subgroup B1 MBLs, comprising 46 reported variants (http://www.lahey.org/Studies/other.asp). They can be divided into the VIM-1 (14), VIM-2 (15), VIM-7 (16), VIM-12 (17), and VIM-13 (18) clusters based on amino acid similarities (19). VIM-1 was first reported in 1999 in Italy (20), with the VIM-2 variant being identified shortly thereafter in France (21) and Italy (22). VIM-4, differing from VIM-1 by one residue (Ser228Arg), was later reported in Pseudomonas aeruginosa isolates in Greece (23). VIM-5 was subsequently identified in Turkey in Klebsiella pneumoniae and Pseudomonas aeruginosa isolates (24, 25) and was later identified in Enterobacter cloacae isolates (26). VIM-38, which differs from VIM-5 by a single substitution (Ala316Val), was recently identified in Pseudomonas aeruginosa isolates in Turkey (27). This conservative substitution at the C terminus (second-to-last residue) is present in ∼50% of VIMs. The greatest variability in amino acid sequence across the five VIM variants is found in the N-terminal leader sequence and, to a lesser extent, in the C-terminal residues (see Fig. S1 in the supplemental material). VIM-2, one of the most widely reported MBLs, shares ∼90% amino acid similarity with the VIM-1 cluster (28). VIM-5 and VIM-38 belong to the VIM-1 cluster and differ from VIM-1 by 5 and 6 residues, respectively (Ala130Lys, His224Leu, Glu225Ala, Ser228Arg, and Lys291Thr, with an additional Ala316Val substitution for VIM-38). Both VIM-5 and VIM-38 contain His224Leu and Ser228Arg substitutions relative to VIM-1; these residues are positioned on the L10 loop and are proposed to influence the substrate specificity of VIM variants (13, 29–32).

Unlike the serine-β-lactamases, as yet, there are no clinically useful MBL inhibitors. The development of broad-spectrum MBL inhibitors is challenging, in part because of structural variation across (and even within) subclasses but also because of the requirement for selective inhibition of bacterial MBLs over human MBL-fold enzymes, some of which have important physiological roles. The continued emergence of new MBL variants with altered substrate selectivity presents a further challenge to inhibitor development. Despite the increased number of reports of VIM variants, structural information is available for only five of these variants (VIM-2, VIM-4, VIM-7, VIM-26, and VIM-31), and biochemical characterization has been carried out for <10 VIM variants (15, 19, 30, 32, 33). Biochemical studies have reported that VIM-5 manifests a level of carbapenemase activity similar to those of VIM-1 and VIM-2 but with greater efficiency toward imipenem than meropenem (26). However, as exemplified by studies with NDM variants (34), it is desirable to compare the activities of MBL variants under the same experimental conditions. To date, the important question of whether all the clinically relevant MBL variants are similarly inhibited has not been addressed.

Here, we report studies on the biochemical and biophysical properties of VIM-4, VIM-5, and VIM-38, in comparison with those of the VIM-1 and VIM-2 enzymes, carried out under the same experimental conditions. We observe modest differences in the kinetic parameters for β-lactamase activities against a variety of β-lactam substrates; however, the results reveal clear differences in the thermal stabilities of the VIM variants, as recently reported for NDM variants (34). Interestingly, the tested variants show clear differences in their inhibition profiles, with one isoquinoline derivative selectively inhibiting VIM-5 and VIM-38 more potently than VIM-1, VIM-2, or VIM-4. Crystallographic studies on VIM-5 reveal structural differences that rationalize the observed differences in inhibition potency.

## MATERIALS AND METHODS

### Cloning and mutagenesis.

DNA encoding full-length VIM-38, lacking its N-terminal periplasmic signaling sequence, was cloned into the pET-28a vector (Novagen) for the production of recombinant protein with an N-terminal His_6_ tag (pET28a-*bla*_VIM-38_). Site-directed mutagenesis (Ala316Val) was performed to generate VIM-5 using pET28a-*bla*_VIM-38_ as a template. Insertion PCR was carried out to incorporate a cleavage site for human rhinovirus 3C (HRV3C) protease into the N terminus of the VIM-5 and VIM-38 sequences. Primers used for mutagenesis are listed in Table S1 in the supplemental material.

For the production of N-terminally His_6_-tagged VIM-1, Escherichia coli codon-optimized VIM-1 coding sequences were inserted into pNIC28-Bsa4 by using standard procedures (35).

### Protein production and purification.

E. coli BL21(DE3) cells were transformed with plasmids encoding VIM-1, VIM-5, and VIM-38 for protein production. The cells were cultured in 2× TY medium supplemented with kanamycin (50 μg/ml), until mid-log phase (optical density at 600 nm [OD_600_] of ∼0.7) was reached. The production of the recombinant proteins was then induced by the addition of 0.1 mM isopropyl-β-d-1-thiogalactopyranoside (IPTG), and the cells were cultured for a further 16 h at 18°C. Cells were harvested by centrifugation (7,000 × *g* for 10 min) and lysed by sonication. Three-step protein purification was carried out using nickel ion affinity chromatography followed by size exclusion chromatography, as previously reported (34). The purified VIM-5 and VIM-38 proteins were incubated overnight at 4°C with His-tagged HRV3C protease or with His-tagged tobacco etch virus (TEV) for VIM-1, to remove the N-terminal His_6_ tag, and further purified by using a second nickel ion affinity column to obtain the untagged enzymes. VIM-2, VIM-4, and C-terminally His_6_-tagged VIM-1 were produced and purified as previously described (19, 36, 37). The purity of the proteins was ascertained by SDS-PAGE; mass spectrometric analysis under both denaturing and nondenaturing conditions was used to verify the masses and metal contents of the purified VIM-5 and VIM-38 enzymes (see Fig. S2 and S3 in the supplemental material).

### Analysis of secondary structure content and melting temperature.

**FIG 2:**
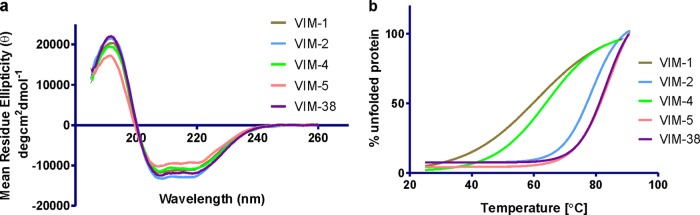
Circular dichroism analyses comparing CD spectra at 25°C (a) and melting temperatures (b) of the tested VIM variants.

Circular dichroism (CD) analyses were carried out using a Chirascan CD spectrophotometer (Applied Photophysics) equipped with a Peltier temperature-controlled cell holder. CD measurements were collected in the range of 185 to 260 nm; spectra were baseline corrected and smoothed using a Savitzky-Golay filter. Data were normalized at 207 nm to account for differences in protein concentrations (38), and the estimation of secondary structure content was performed with Dichro Web (39) using the CONTIN Analysis Programme (reference set 6) (40). Melting temperatures (*T_m_*) of the recombinant enzymes were determined by monitoring temperature-induced changes in the CD signal at 222 nm. The temperature was increased by 1°C per min, and the CD signal was recorded at temperatures ranging from 25°C to 92°C. The samples were then cooled from 92°C to 25°C at the same rate, and a CD spectrum of the refolded proteins was determined. The activity of the enzymes was recorded before thermal denaturation and after refolding by monitoring nitrocefin hydrolysis. The thermal denaturation data were fitted to a Boltzmann sigmoidal curve by using GraphPad Prism 5.0 software. CD spectra and thermal denaturation curves are shown in Fig. 2 and in Tables S2 and S3 in the supplemental material.

### Determination of kinetic parameters.

The hydrolysis of a representative panel of β-lactam substrates was monitored at their respective absorbance wavelengths. The wavelengths and extinction coefficients used were previously described (34). The assays were carried out using 50 mM HEPES buffer (pH 7.2) supplemented with 1 μg/ml bovine serum albumin (BSA), 1 μM ZnSO_4_, and 0.01% Triton X-100. In the case of VIM-1, C-terminally His_6_-tagged VIM-1 was used for kinetic analyses. Note that no differences were observed between the activities of untagged VIM-1 and C-terminally His_6_-tagged VIM-1 for the selected antibiotics (data not shown). The initial rates were obtained from the changes in absorbance at various substrate concentrations. Steady-state kinetic parameters (*K_m_* and *k*_cat_) were determined by fitting the initial velocity data to the Michaelis-Menten equation using GraphPad Prism 5.01 software (see Table S4 in the supplemental material).

### Inhibition assays.

Inhibitors were prepared as reported previously (36, 41). Enzyme-mediated hydrolysis of nitrocefin was monitored by determining the changes in absorbance at 495 nm, as previously described (36, 37). Residual activities were first determined at a 100 μM inhibitor concentration. For the determination of 50% inhibitory concentrations (IC_50_s), the reporter substrate (nitrocefin) was used at near-*K_m_* values, and the enzyme was preincubated with the inhibitors for 10 min at room temperature prior to the addition of the substrate (42). The residual activities were obtained at increasing inhibitor concentrations (0.2 to 2,000 μM); the data were analyzed using GraphPad Prism 5.01 software.

### NMR binding assays.

Binding assays were carried out using ^1^H-edited Carr-Purcell-Meiboom-Gill (CPMG) nuclear magnetic resonance (NMR) analyses. Spectra were recorded by using a Bruker AVIII 600-MHz NMR spectrometer equipped with a BB-F/^1^H Prodigy N_2_ cryoprobe using 3-mm Match NMR tubes (Cortectnet). The PROJECT-CPMG sequence (90°*x*-[τ-180°*y*-τ-90°*y*-τ-180°*y*-τ]*n*-acq) was applied, and water suppression was achieved by presaturation. Data were collected with a sweep width of 12,019 Hz, an acquisition time of 2.7 s, and a filter width of 125,000 s. Assays were conducted using 50 mM Tris-D_11_ (pH 7.5) supplemented with 0.02% NaN_3_ in 90% H_2_O and 10% D_2_O. Equilibrium dissociation constant (*K_D_*) measurements for the reporter ligand (50 μM thiomandelic acid) and inhibitors (0 to 300 μM) were carried out as previously reported (43). Data were processed with Bruker 3.1 software and fitted by using Origin Pro8.5.1 (see Table S5 in the supplemental material).

### VIM-5 crystallography.

Purified VIM-5 was crystallized using the sitting-drop vapor diffusion method with 96-well, 3-subwell Intelliplates (Art Robbins). Drops of 300 nl were obtained by adding a protein solution (23 mg/ml) and the reservoir buffer at a 2:1 ratio (protein/reservoir). Crystals formed in wells containing 0.1 M Tris buffer (pH 8.5), 25% polyethylene glycol 3350 (PEG 3350), and 0.2 M NaCl. A cryoprotectant solution was prepared by diluting the well solution with glycerol to a final concentration of 25% (vol/vol) glycerol. An ∼10× drop volume of cryoprotectant was then added to the top of a drop containing crystals, and the crystals were harvested using a nylon loop, followed by plunging into liquid nitrogen. Data were then collected for a single crystal at the Diamond Light Source synchrotron beamline. Data were processed by using XDS and CCP4-SCALA in XIA2 (44–46). Initial phases were obtained by molecular replacement (MR) (47) using the PHASER (48) subroutine within PHENIX (49, 50), with the structure of VIM-4 (Protein Data Bank [PDB] accession number 2WRS) (51) as the search model. Crystallographic structure refinement was carried out by iterative rounds of model building using WinCoot (52) and maximum likelihood restrained refinement using PHENIX. Data collection and refinement statistics are given in Table S6 in the supplemental material.

### Protein structure accession number.

Coordinates and structure factors for VIM-5 have been deposited in the PDB under accession number 5A87.

## RESULTS

Recombinant VIM-1, VIM-2, VIM-4, VIM-5, and VIM-38 were efficiently produced and purified to near homogeneity as determined by SDS-PAGE analysis (see Fig. S2 in the supplemental material). The molecular masses of the recombinant proteins as determined by liquid chromatography-mass spectrometry (LC-MS) analyses were in close agreement with the theoretical values. Data from nondenaturing electrospray ionization mass spectrometric analyses validated the identities of the VIM-5 and VIM-38 variants and were consistent with the binding of two metal (zinc) ions for each of the purified proteins (see Fig. S3 in the supplemental material).

### Biophysical characterization.

To compare the secondary structural content of the VIM variants, far-UV circular dichroism (CD) analyses were carried out on the untagged recombinant proteins. All five VIM variants exhibited very similar CD spectra, with a slight deviation in ellipticity at lower wavelengths, indicating that the amino acid substitutions do not cause major perturbations in secondary structure (Fig. 2a).

We then carried out temperature-dependent CD spectroscopy to investigate the relative thermal stability of the VIM variants, because some substitutions in VIM and NDM MBLs have been associated with variations in protein stability (28, 34). With the exception of VIM-1 and VIM-4, the CD spectra of the cooled samples were nearly identical to those obtained before heating. For all the tested VIM variants, the refolded samples retained substantial activity (<2-fold differences as judged by *k*_cat_/*K_m_* values), indicating that proteins regained their native structure upon refolding (see Table S3 in the supplemental material). The observed reversibility allowed further thermodynamic analysis to obtain melting temperature (*T_m_*) values. Significant differences in the *T_m_* values of the five VIM variants were observed. VIM-5 and VIM-38 were the most stable variants, with an apparent *T_m_* of >83°C, which is ∼5°C higher than that determined for VIM-2 (78°C). VIM-1 and VIM-4 were the least stable of the tested variants, with apparent *T_m_* values of 60°C and 64°C, respectively, which are >20°C lower than those of VIM-5 and VIM-38 and >14°C lower than that of VIM-2 (Fig. 2b).

### Functional properties of VIM variants.

The hydrolytic activities of the VIM variants against a representative set of β-lactam substrates were then compared under similar assay conditions. Overall, the tested variants showed relatively small differences in their catalytic efficiencies toward the tested β-lactam substrates, with VIM-5 and VIM-38 showing very similar kinetic parameters (Table 1). In some cases, differences in individual kinetic parameters were observed, sometimes contributing to changes (albeit relatively small ones) in the overall hydrolytic efficiency as defined by *k*_cat_/*K_m_* values. For instance, for VIM-1, the *K_m_* value for meropenem was >6-fold higher than that for VIM-2, indicating a lower affinity for this substrate. This difference was, however, counterbalanced by high *k*_cat_ values for VIM-1 (>7-fold higher than those for VIM-2), resulting in similar *k*_cat_/*K_m_* values. Despite VIM-1 and VIM-2 variants having similar *K_m_* values for imipenem (likely within experimental error), VIM-2 exhibited higher *k*_cat_ values, resulting in a 6-fold increase in *k*_cat_/*K_m_* value compared to that for VIM-1. Relatively high apparent *K_m_* values (>300 μM) were recorded for all VIM variants with ampicillin, indicating a relatively low affinity for this penicillin. Notably, VIM-5 and VIM-38 had >10-fold-higher *k*_cat_ values for cefoxitin than did VIM-1, resulting in a >7-fold increase in *k*_cat_/*K_m_* values. Cephalothin and nitrocefin were efficiently hydrolyzed by all the tested VIM variants, recording the highest *k*_cat_/*K_m_* values compared to those with the other β-lactam substrates. Ceftazidime was a poor substrate for the variants, with *k*_cat_/*K_m_* values being <0.1 s^−1^/μM for the tested VIM variants. Apparent kinetic parameters were reported for ceftazidime and ampicillin hydrolysis, since the initial velocity remained proportional to the substrate concentration up to 300 μM (see Table S4 in the supplemental material).

**TABLE 1:** Kinetic parameters for VIM-1, VIM-2, VIM-5, and VIM-38 with a representative panel of β-lactam substrates

Substrate	Mean *K_m_* (μM) ± SD^*a*^				*k*_cat_ (s^−1^)				*k*_cat_/*K_m_* (s^−1^/μM)			
	VIM-1^*b*^	VIM-2	VIM-5	VIM-38	VIM-1	VIM-2	VIM-5	VIM-38	VIM-1	VIM-2	VIM-5	VIM-38
Meropenem	130 ± 20	20 ± 4	70 ± 10	30 ± 4	50	7	20	10	0.40	0.30	0.30	0.30
Imipenem	60 ± 10	30 ± 4	60 ± 6	150 ± 20	30	100	60	90	0.50	3.30	1.00	0.60
Ampicillin	>1,000	>500	>1,000	380 ± 80	120	180	230	170	0.10	0.40	0.20	0.40
Cefoxitin	170 ± 50	40 ± 10	320 ± 60	230 ± 60	30	50	460	340	0.20	1.30	1.40	1.50
Ceftazidime	180 ± 60	120 ± 20	340 ± 80	430 ± 140	3	2	4	4	0.02	0.02	0.01	0.01
Cephalothin	140 ± 10	50 ± 6	100 ± 8	60 ± 8	450	210	200	120	3.20	4.20	2.00	2.00
Nitrocefin	15 ± 1	50 ± 5	50 ± 5	70 ± 10	130	510	420	720	8.70	10.2	8.40	10.3

a *K_m_* values are reported as the means of data from three independent measurements ± standard deviations. Apparent kinetic parameters are reported for ampicillin and ceftazidime hydrolysis. Standard deviation values for *k*_cat_ did not exceed 10%.

b C-terminally His_6_-tagged VIM-1 was used for kinetic analyses (36).

### Inhibition of VIM variants.

**FIG 3:**
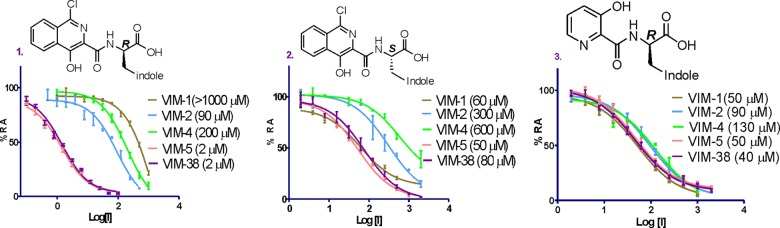
Inhibition of VIM variants by selected inhibitors. RA, residual activity.

To investigate if the VIM variants might manifest different degrees of inhibition, we screened a set of compounds containing different potential metal-chelating motifs (36, 41), including isoquinolines and pyridine-2-carboxylates, to compare their inhibitory effects on the VIM variants. An interesting result to emerge from this work was that the isoquinoline derivative (compound 1) is a substantially better inhibitor of VIM-5 and VIM-38 (IC_50_ = 2 μM) by >500-fold than VIM-1 (IC_50_ of >1 mM). VIM-2 and VIM-4 also had >40-fold-higher IC_50_s (IC_50_s of 90 μM and 200 μM, respectively) with inhibitor 1 than did VIM-5 and VIM-38 (Table 2). The configuration of the stereocenter of the inhibitor side chain is important with respect to the degree of inhibition, with the (*R*)-enantiomer (compound 1) being a more potent inhibitor than the (*S*)-enantiomer (compound 2) for all the variants, except for VIM-1, for which the (*S*)-enantiomer (compound 2) was a more potent inhibitor. The isoquinoline ring system is also apparently important, as the pyridine-2-carboxylate derivative (compound 3) with an (*R*)-tryptophan side chain analogous to that of compound 1 inhibited all VIM variants similarly and with only modest potency (Fig. 3). Interestingly, VIM-4 showed IC_50_s similar to those of VIM-2 for the tested compounds despite differing from VIM-1 by only one residue (Ser228 in VIM-1 and Arg228 in VIM-4).

**TABLE 2:**
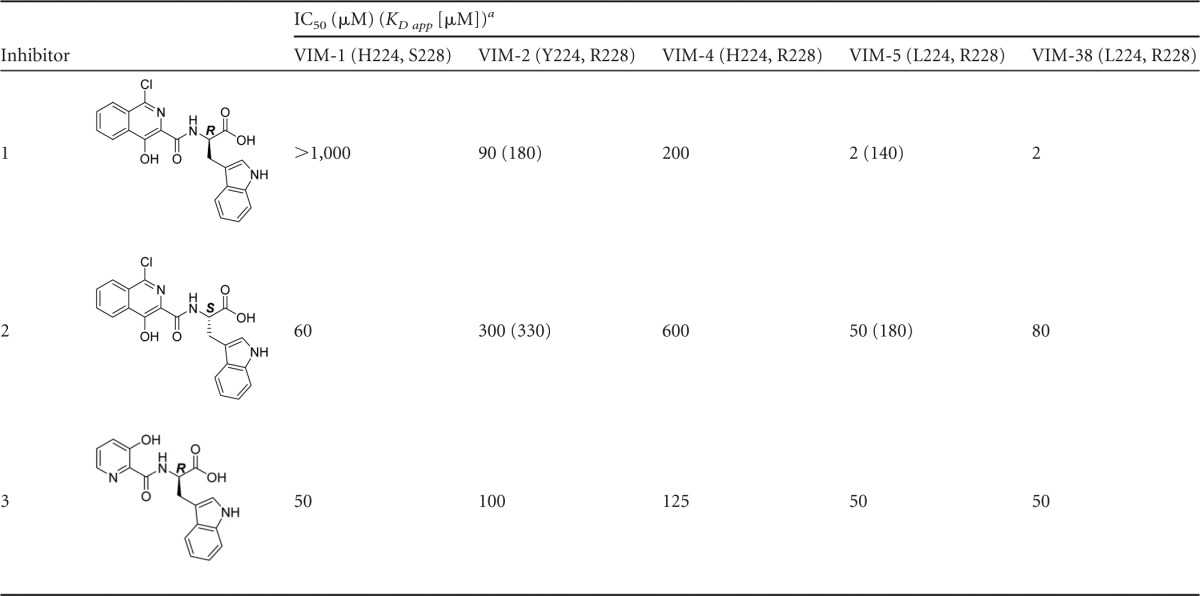
Inhibition of VIM variants by selected isoquinoline and pyridine-2-carboxylates

^a^ IC_50_ determinations were performed in triplicate over a range of inhibitor concentrations from 0.2 to 2,000 μM. *K_D app_* values were determined by ^1^H CPMG NMR experiments (see Table S5 in the supplemental material).

To validate the inhibition results, we carried out ^1^H CPMG NMR binding assays (43). The NMR analyses showed a similar trend, with both isoquinoline inhibitors 1 and 2 binding more strongly to VIM-5 than to VIM-2 (see Table S5 in the supplemental material). The (*R*)-enantiomer (compound 1) was also shown to be a stronger binder to VIM-2 (apparent *K_D_* [*K_D app_*] = 180 μM) than the (*S*)-enantiomer (compound 2) (*K_D app_* = 330 μM), highlighting the importance of the configuration of the side-chain stereocenter in the potency of inhibition.

### Crystallographic analysis.

**FIG 4:**
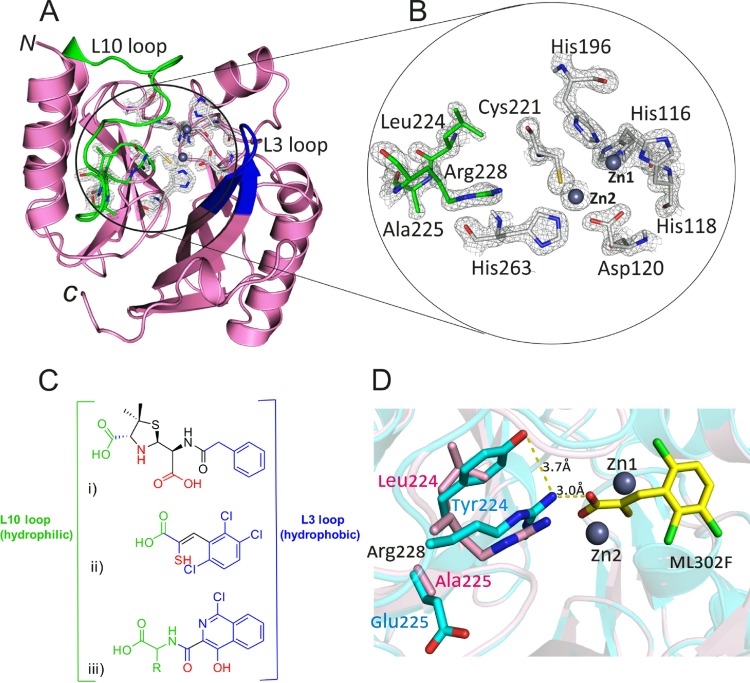
(A) Ribbon representation of the VIM-5 crystal structure (PDB accession number 5A87) showing its overall fold. Metal binding residues are shown in white, and zinc ions are shown as gray spheres. The L3 and L10 loops are highlighted in blue and green, respectively. (B) Closeup view of Leu224, Ala225, and Arg228 residues within the VIM-5 active site. Observed electron density (2*F_o_* − *F_c_* contoured to 1 σ) is shown as gray mesh. (C) Comparison of structures for a hydrolyzed benzylpenicillin (i), the thioenolate inhibitor ML302F (ii), and an isoquinoline inhibitor (iii) showing possible interactions with VIM MBLs. The green atoms likely interact with the hydrophobic L10 loop, while the blue atoms indicate groups likely to interact with the hydrophobic L3 loop. The atoms in red are proposed to interact with the metal center of the enzyme. (D) Superimposition of the VIM-2 (cyan) (PDB accession number 4PVO) and VIM-5 (pink) (PDB accession number 5A87) structures showing the effect of substitutions at residues 224 and 225, as observed for VIM-2 and VIM-5, and showing the binding mode of the thioenolate inhibitor ML302F (yellow) in the VIM-2 active site (54).

To explore the structural features contributing to the observed differences in inhibition of the VIM variants, a crystal structure of recombinant VIM-5 was determined to a 1.5-Å resolution (P2_1_ space group), having 2 molecules per asymmetric unit. The overall fold of VIM-5 was almost identical to that described for the previously reported VIM-2 structure (PDB accession number 4BZ3), with a root mean square distance (RMSD) of 0.206 Å. The overall fold of VIM-5 has the canonical MBL αβ/βα sandwich structure, with two zinc(II) ions (3.5 Å apart) being bound in the active site located in a shallow cleft formed by the interface of the two β-sheets (Fig. 4A). The proposed nucleophilic hydroxide/water molecule bridges between the two zinc(II) ions and is positioned 1.9 Å and 2.0 Å from Zn1 and Zn2, respectively. The zinc(II) ion coordination geometry is similar to the coordination observed for VIM-2 (Fig. 4B) (53). Despite having identical sequences, the VIM-5 L3 loop was closer to the active site than observed for VIM-2, as was reported previously for VIM-4 and VIM-31 (19, 32). It is also notable that a 4.8-Å shift in the carbonyl oxygen position of Ala231 was observed in comparison to the VIM-2 structure (PDB accession number 4BZ3) (see Fig. S4 in the supplemental material).

## DISCUSSION

Overall, the kinetic results reveal relatively small differences in the catalytic efficiencies of the tested VIM variants (Table 1), the biological relevance of which, if any, requires further investigation. VIM-5 was found to have activity toward carbapenems similar that of VIM-1 and VIM-2, as was previously proposed (26). VIM-5 and VIM-38 display very similar kinetic parameters, as expected, given that they differ by 1 residue at position 316 (Ala and Val in VIM-5 and VIM-38, respectively). This substitution at the C terminus of the enzymes is conserved and present in ∼50% of the reported VIM variants (http://www.lahey.org/studies/other.asp#).

The hydrolytic efficiencies presented here are in agreement with those reported previously for some of the substrates (e.g., VIM-2 hydrolyzes imipenem better than meropenem) (21) but differ with regard to other substrates; e.g., in our study, VIM-5 hydrolyzes ceftazidime with a 10-fold-higher efficiency than that previously reported (26). The discrepancies observed may be attributed to differences in enzyme preparation and assay conditions, emphasizing the need to carry out comparative kinetic studies under similar experimental conditions (see Table S7 in the supplemental material for a comparison of our data with data from previous studies).

The VIM variants tested in this study exhibited similar CD spectra, implying that their secondary structures are not substantially affected by the differences in their sequences (Fig. 2a). However, marked differences in the thermal stabilities of the variants were observed, with VIM-5 and VIM-38 having the highest melting temperatures (84°C and 83°C, respectively) compared to VIM-2 (78°C). VIM-1 (60°C) and VIM-4 (64°C) showed significantly lower melting temperatures (Fig. 2b). These results are in agreement with previously reported low melting temperatures for VIM-4 (19). The results showing increased thermal stability of at least some VIM variants is interesting, as we have observed similar trends with NDM variants (34). As proposed in the case of the NDM variants, it is possible that the increased thermal stabilities of emergent MBL variants could reflect increased cellular lifetimes, directly due to either the improved thermal stability or a reduced propensity for proteolysis or aggregation (34). Thus, although “evolutionary drift” cannot be ruled out and further work is required, the increased thermal stability of some MBL variants presents an interesting line of investigation.

Unlike the relatively small differences in kinetic parameters observed, very clear differences were observed for the inhibition of different VIM variants with some (but not all) types of inhibitors tested (Fig. 3). These differences are most clearly exemplified by the relatively low IC_50_s reported for VIM-5 and VIM-38 with isoquinoline compound 1 compared to those for VIM-1, VIM-2, and VIM-4 (Table 2). A crystal structure of VIM-5 reveals interesting active-site differences compared to VIM-2 and provides some possible insights into the differences in the catalytic and inhibitory profiles of the VIM variants (Fig. 4).

The substitutions at residue 224 (His, Tyr, and Leu in VIM-1/VIM-4, VIM-2, and VIM-5/VIM-38, respectively), which is located on the L10 loop, likely contribute to the differential inhibition profiles observed. Comparison of the crystal structures of the VIM variants reveals that replacement of Tyr224, as in VIM-2, with Leu224, as in VIM-5 and VIM-38, likely provides a more spacious active site that may better accommodate the bulky tryptophan side chains of the tested inhibitors, as reflected by the increased inhibition of VIM-5 and VIM-38 by inhibitors 1 and 2. The smaller side chain of Leu224 in VIM-5 also enables a “flipping” of the main-chain carbonyl of Ala231, altering the entrance to the active site and likely affecting inhibitor binding. Notably, the main-chain flipping of Ala231 is also observed for other enzymes with Leu224, such as VIM-26 (30) (see Fig. S4 in the supplemental material).

The bicyclic isoquinoline ring system of inhibitors 1 and 2 may interact with the hydrophobic residues on the mobile L3 loop (residues 60 to 66) (15), leading to better binding than with pyridine-2-carboxylate inhibitor 3 for VIM-5 and VIM-38. It is proposed that VIM-2 residue Glu225 forms electrostatic interactions that rigidify and partially neutralize the side chain of Arg228, which interacts with the carboxylate of β-lactam substrates (15, 19). The replacement of Glu225 with Ala225, as in VIM-5 and VIM-38, may enable more flexibility of the Arg228 side chain, contributing to the observed differences in substrate/inhibitor binding. Additionally, the hydrogen bond formed between the Glu225 side chain and the main chain of Leu265 is disrupted in the VIM-5 structure with the replacement of Glu225 with Ala225. This may also have implications for substrate-inhibitor interactions.

The overall results reveal that the tested VIM variants show relatively small differences in their catalytic efficiencies with the tested substrates, suggesting that changes in substrate selectivity are not their sole evolutionary driving force, as was recently proposed for NDM variants (34). We observed a marked difference in thermal stability for the VIM variants, as observed for NDM variants, suggesting that this may reflect a selection pressure (34). Perhaps most importantly, the results reveal that clinically observed MBL variants can manifest different inhibition profiles, a factor that we propose should be taken into account at an early stage in inhibitor development programs.

## Supplementary Material

Supplemental material
