# Supplementary material for: Comparison of Verona Integron-Borne Metallo-β-Lactamase (VIM) Variants Reveals Differences in Stability and Inhibition Profiles
Source: Antimicrob Agents Chemother. 2016 Feb 26;60(3):1377–84. doi: 10.1128/AAC.01768-15 (PMC4775916; doi:10.1128/AAC.01768-15)
Supplement: Supplemental material [file supp_60_3_1377__index.html]

Supplemental material 

# Comparison of Verona Integron-Borne Metallo-β-lactamase Variants Reveals Differences in Stability and Inhibition Profiles

## Supplemental material

- Supplemental file 1 -

  Supplemental Figures S1 to S4 and Tables S1 to S7

  PDF, 5.5M
