# Supplementary material for: Comparison of Verona Integron-Borne Metallo-β-Lactamase (VIM) Variants Reveals Differences in Stability and Inhibition Profiles
Source: Antimicrob Agents Chemother. 2016 Feb 26;60(3):1377–84. doi: 10.1128/AAC.01768-15 (PMC4775916; doi:10.1128/AAC.01768-15)
Supplement: Supplemental material [file AAC.01768-15_zac003164892so1.pdf]

Supplementary Material

Comparison of Verona Integron-Borne Metallo-β-lactamase Variants Reveals Differences in Stability and Inhibition Profiles

Anne Makena, Azer Ö. Düzgün, Jürgen Brem, Michael A. McDonough, Anna M. Rydzik, Martine I. Abboud, Ayşegül Saral, Ayşegül Ç. Çiçek, Cemal Sandalli and Christopher J. Schofield

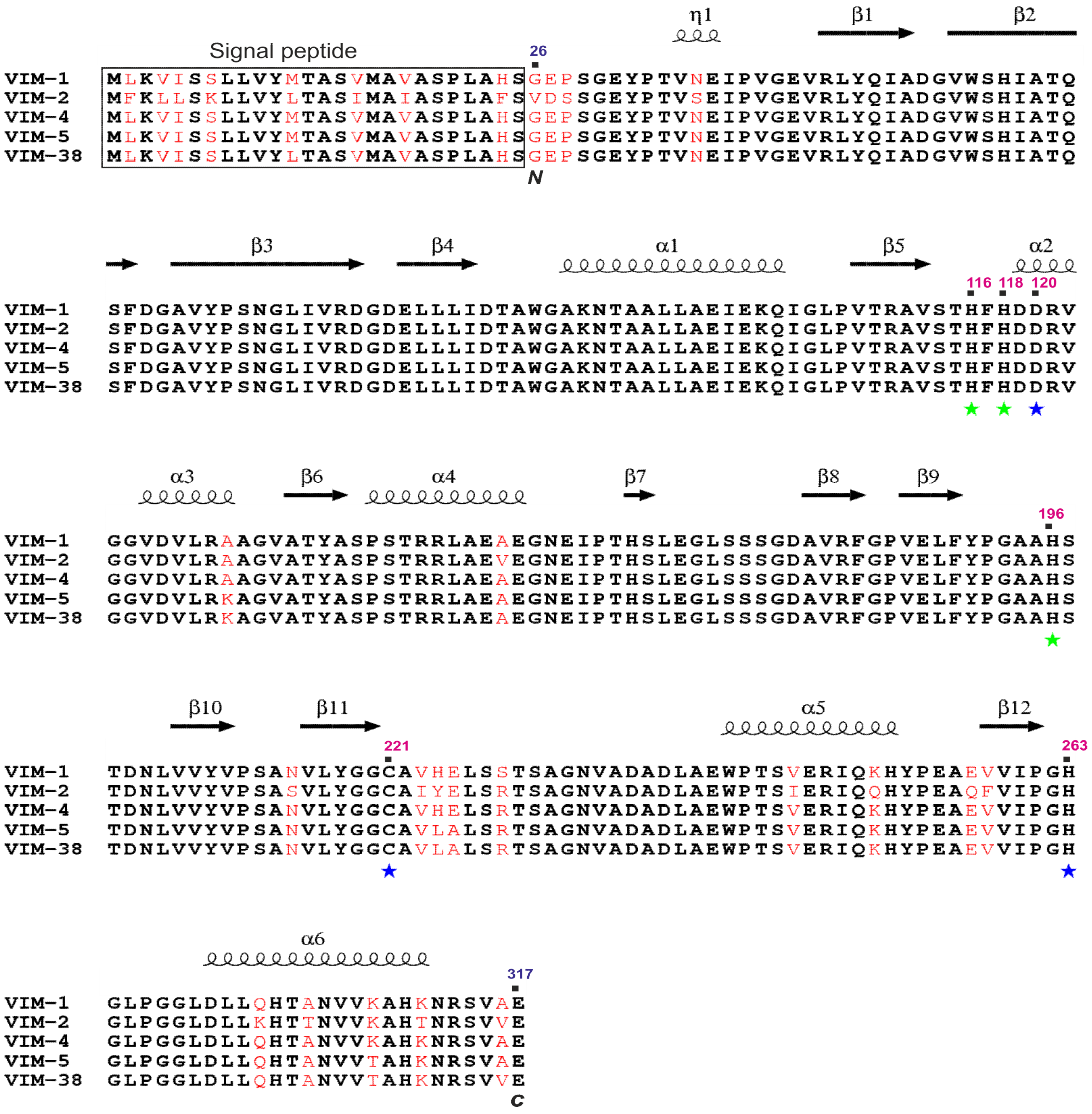

Figure S1: Sequence alignment of the VIM-1, VIM-2, VIM-5 and VIM-38 MBLs. Amino acid sequence alignment of the tested VIM variants generated using ESPript 3.0 (1). The secondary structure assignments are based on the VIM-5 structure (PDB code 5A87). The green and blue stars indicate the active site residues (labelled in magenta) interacting with the Zn1 and Zn2 ions respectively. Residues in red indicate the amino acid differences between the tested variants. The *N*- and *C*- terminal residues of the mature enzymes are labelled 26 and 317 respectively.

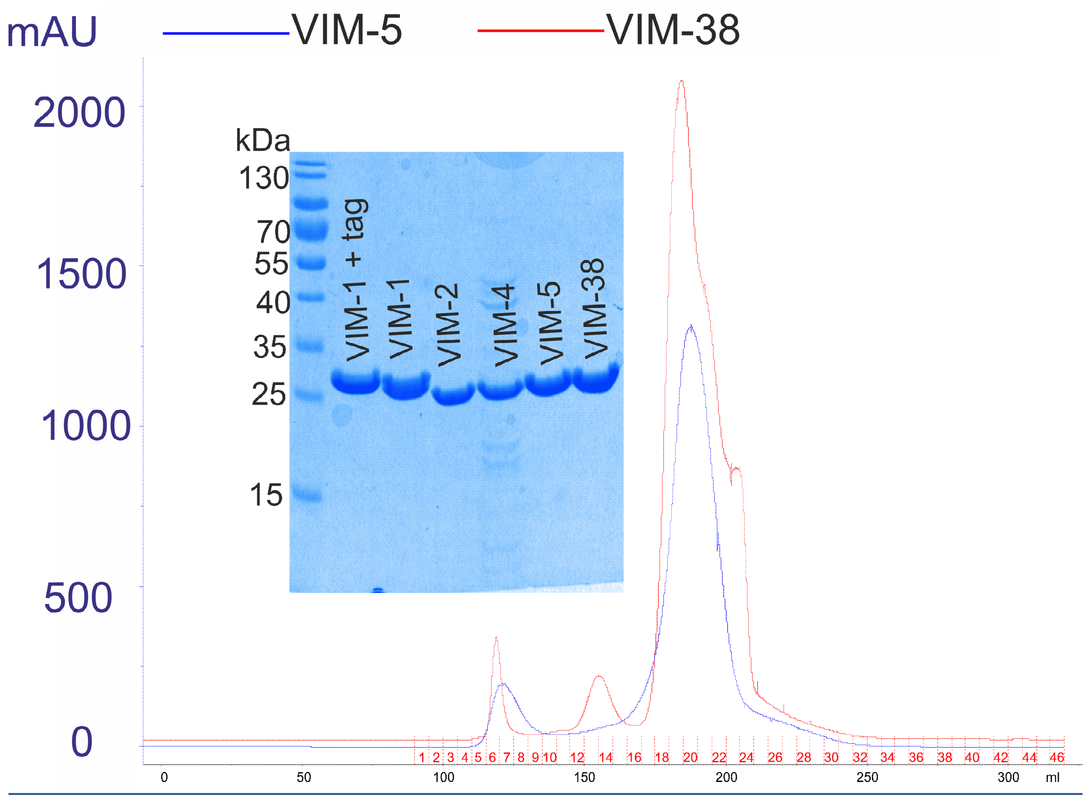

Figure S2: VIM variants protein production and purification. Gel filtration chromatograms for VIM-5 (blue) and VIM-38 (red). **Inset**: SDS-PAGE analysis of untagged VIM-1, VIM-2, VIM-4, VIM-5 and VIM-38 after the cleavage of the *N*-terminal His_6-tag, showing single protein bands with >95% purity at ~25kDa molecular weight. PageRuler Prestained Protein Ladder was used as the molecular mass marker (see Materials and Methods for details).

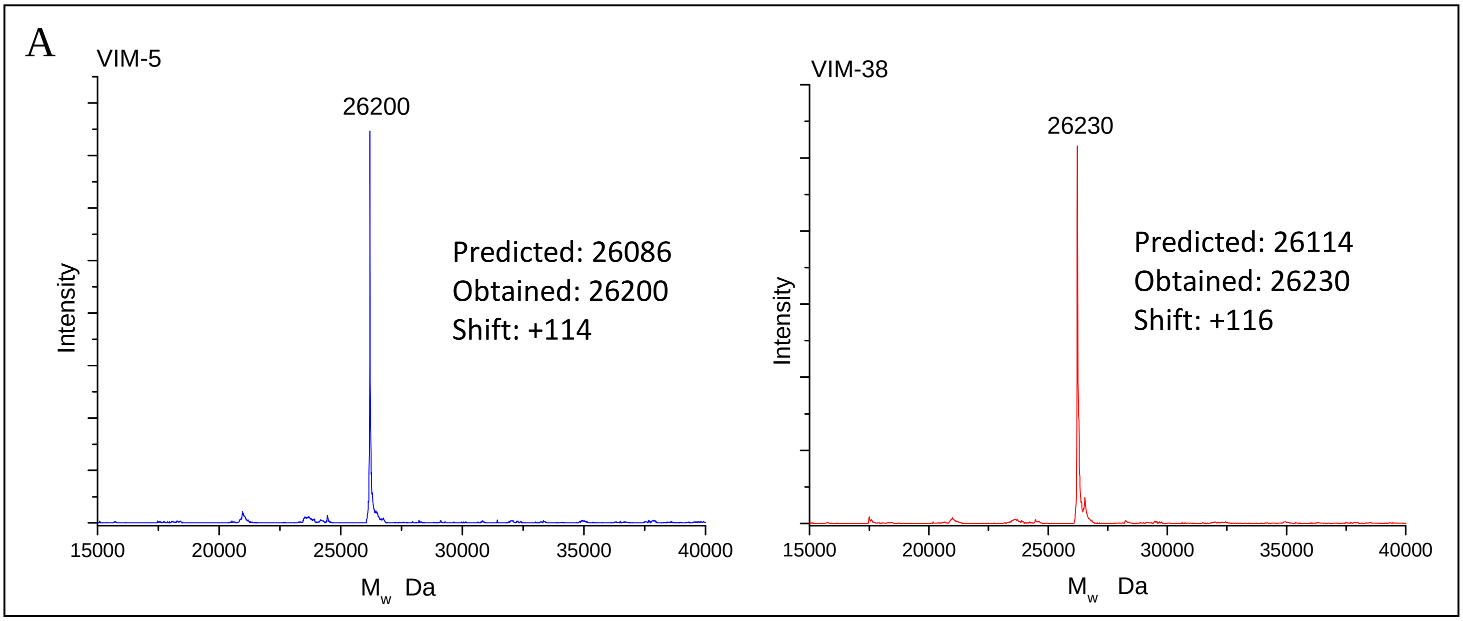

B

**Enzyme**	**Obtained values**	**Theoretical Values**
VIM-1 (C-His6 tagged)	26273	26275
VIM-1 (untagged)	25410	25409
VIM-2	25670	25669
VIM-4	25392	25391
VIM-5	26087	26086
VIM-38	25115	25114

Figure S3: Mass spectrometric analyses of VIM variants. A; Non-denaturing mass spectrometric analyses showing deconvoluted spectra for VIM-5 and VIM-38. The difference between the obtained and predicted mass (shift) corresponds to the presence of two zinc ions (130 ± 14 Da). **B;** The list indicates the masses of the recombinant proteins as determined by LC-MS analyses under dematuring conditions. The observed masses were verified by comparison with the predicted masses obtained using the ExPasy ProtParam tool (http://web.expasy.org/protparam/).

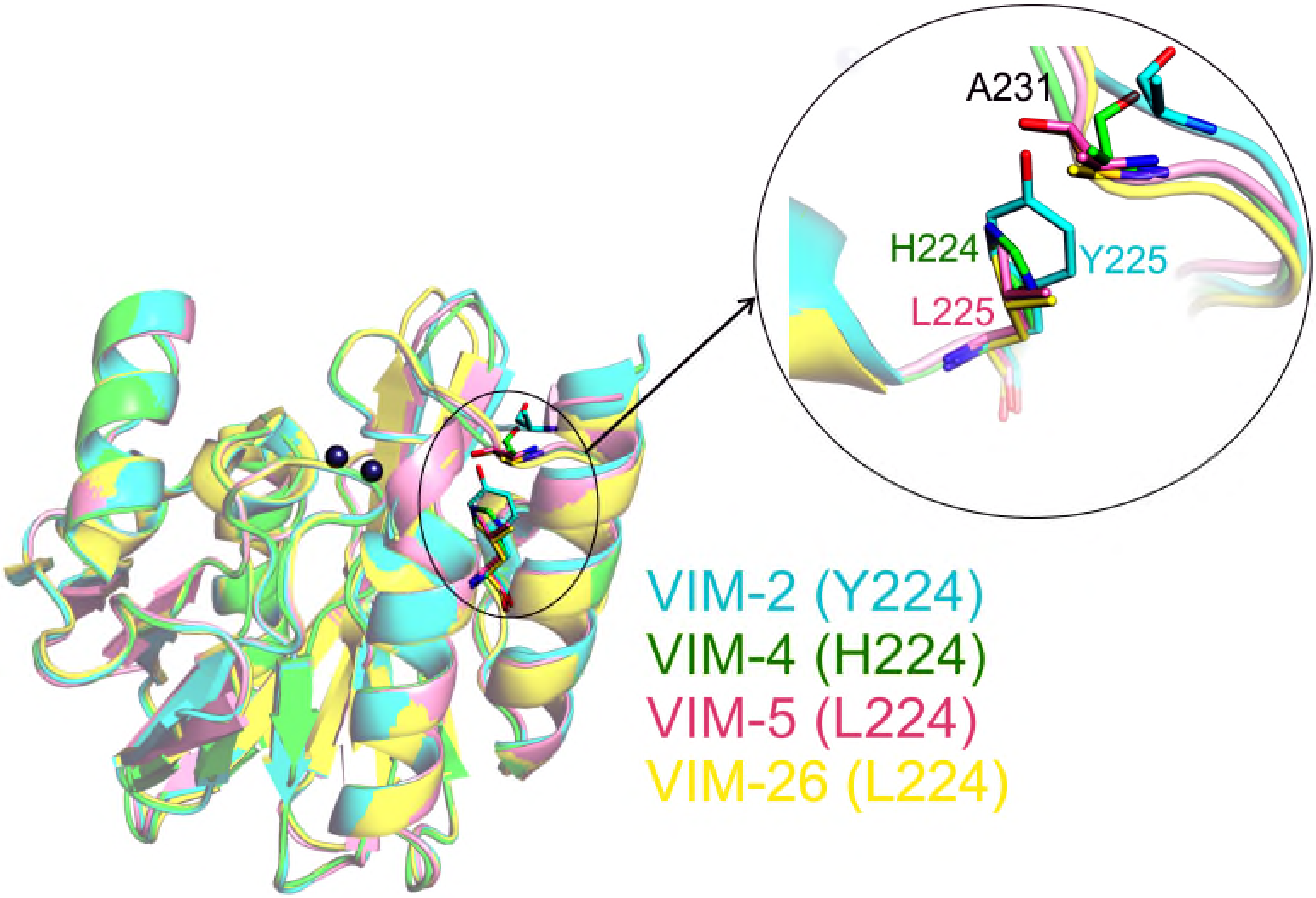

Figure S4: **Comparison of VIM variants structures**. View from a structural overlay of VIM-5 (pink) (PDB code: 5A87) and VIM-2 (cyan) (PDB code: 4BZ3), VIM-4 (green) (PDB code: 2WRS) and VIM-26 (yellow) (PDB code: 4UWO) structures showing similarities of the overall folds (2, 3). **Inset**: Close-up view of the structural overlay showing a shift in carbonyl oxygen position of Ala231 in VIM-5 and VIM-26 compared to VIM-2 and VIM-4, concomitant with a main chain ‘flip’ of the Ala231 carboxyl group, apparently influenced by the sterics of different residues at position 224 (Tyr in VIM-2, His in VIM-4 and Leu in VIM-5 and VIM-26).

Table S1: Primers used for mutagenesis.

a) Site directed mutagenesis primers for the substitution Ala316Val

Forward	5’-CACAAAAATCGCTCAGTCGCCGAG-‘3
Reverse	5’-CTCGGCGACTGAGCGATTTTTGTG-3’

b) Primers for insertion of a human rhinovirus 3C (HRV3C) protease cleavage site

Forward	5’-GGCTAGCATGACTGAATTCCTGGAAGTGCTGTTTCAGGGCCCGAGTC CGTTAGCCCATTC-3’
Reverse	5’-GAATGGGCTAACGGACTCGGGCCCTGAAACAGCACTTCCAGGAATTC AGTCATGCTAGCC-3’

Table S2. Comparison of protein spectra as determined by CD analyses and hydrolytic activity before and after thermal denaturation (See Materials and Methods for details).

Enzyme	Protein Spectra	Pre and post CD kinetics
VIM-1	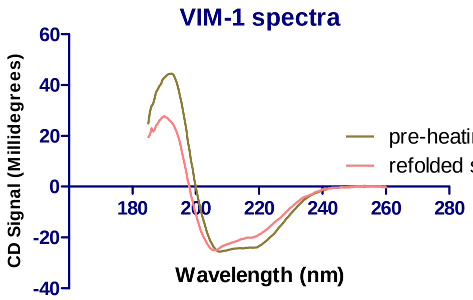	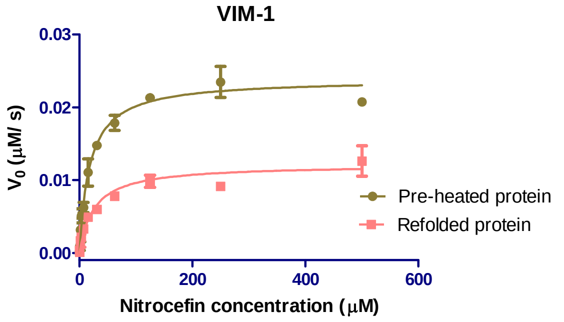
VIM-2	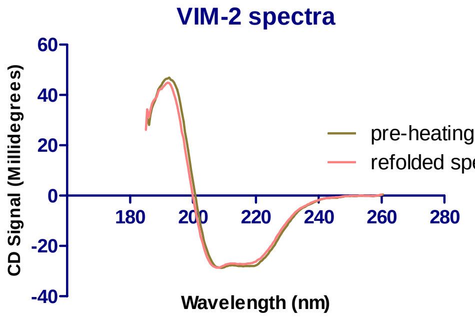	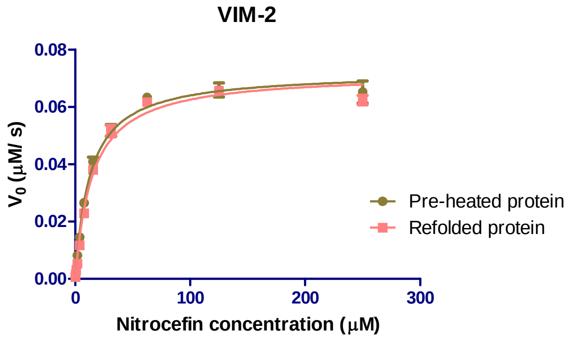
VIM-4	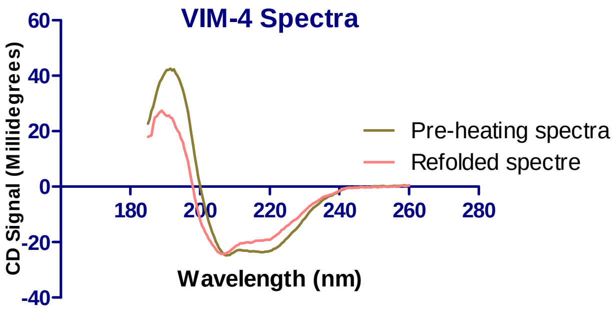	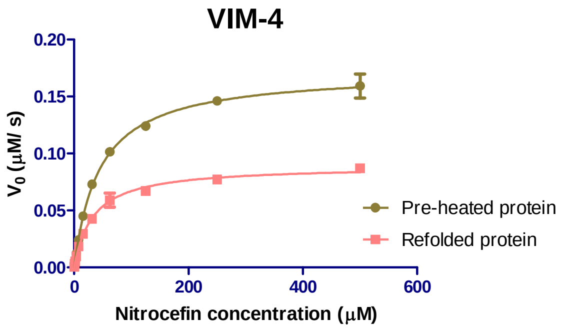
VIM-5	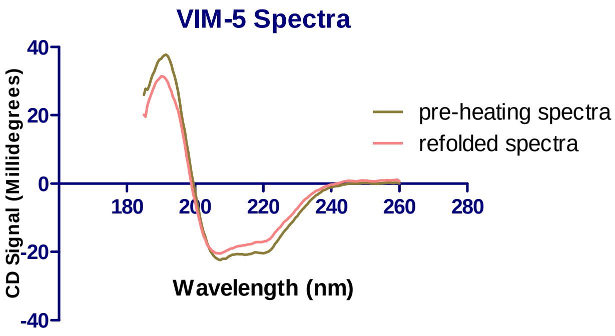	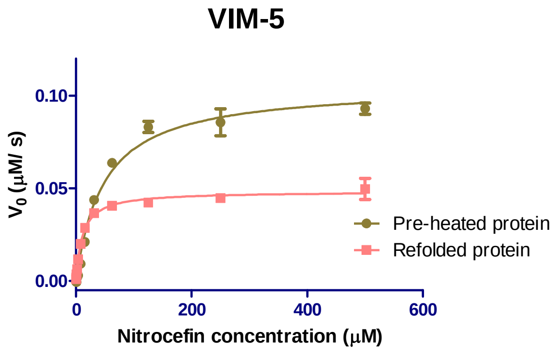

VIM-38

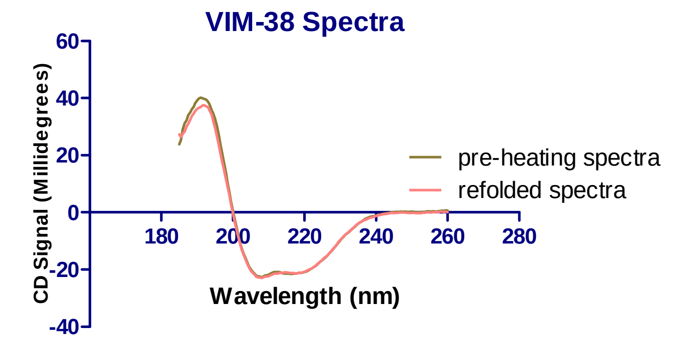

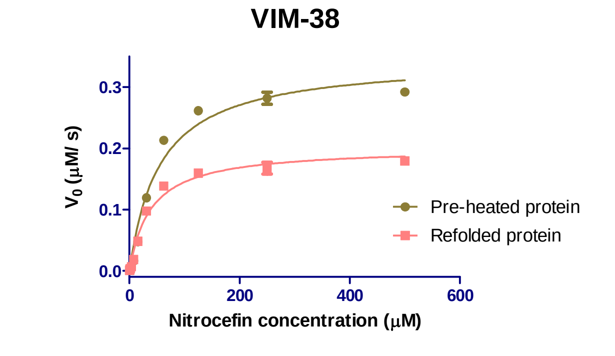

Table S3: Kinetic parameters of VIM variants before thermal denaturation and after renaturation.

	Pre-heating			After refolding		
	*K_m (µM)	k_{cat} (s^{-1})	k_{cat}/K_m (s^{-1}/µM)	K_m (µM)	k_{cat} (s^{-1})	k_{cat}/K_m (s^{-1}/µM)
**VIM-1**	20 ± 2	25	1	26 ± 3	12	0.5
**VIM-2**	13 ± 1	290	22	15 ± 1	290	19
**VIM-4**	45 ± 1	340	8	30 ± 2	180	5
**VIM-5**	45 ± 3	210	5	11 ± 1	100	9
**VIM-38**	55 ± 6	690	13	40 ± 4	100	10

*K_m values are reported as the means of three independent measurements ± standard deviation. Standard deviation values for k_{cat} did not exceed 10 %.

Table S4: Steady state kinetic analyses of the tested VIM variants with representative β-lactam substrates.

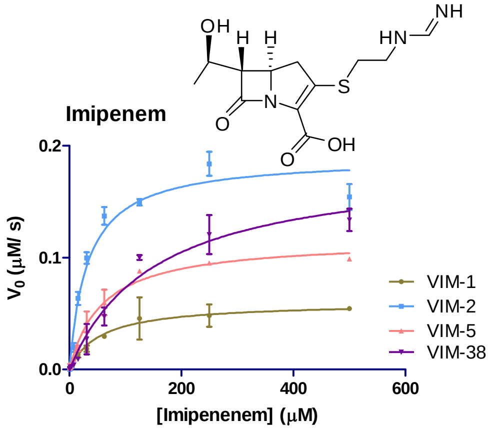

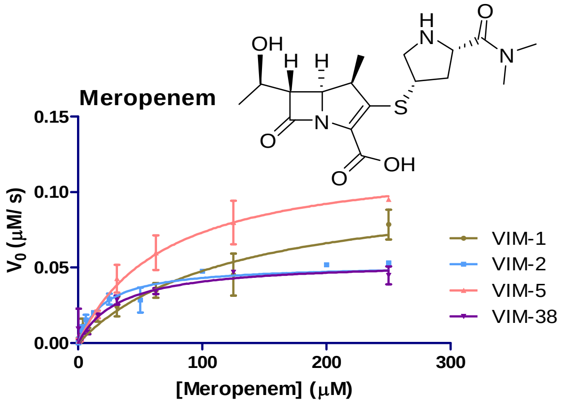

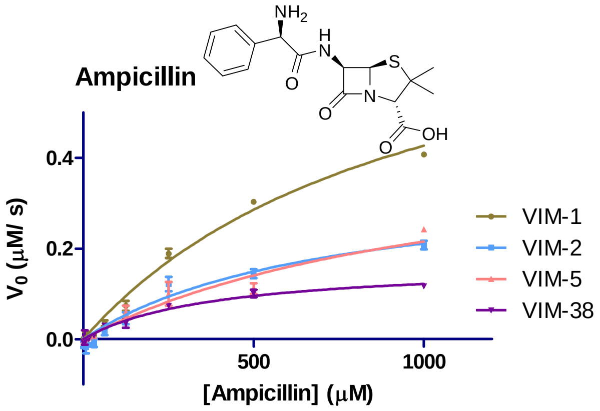

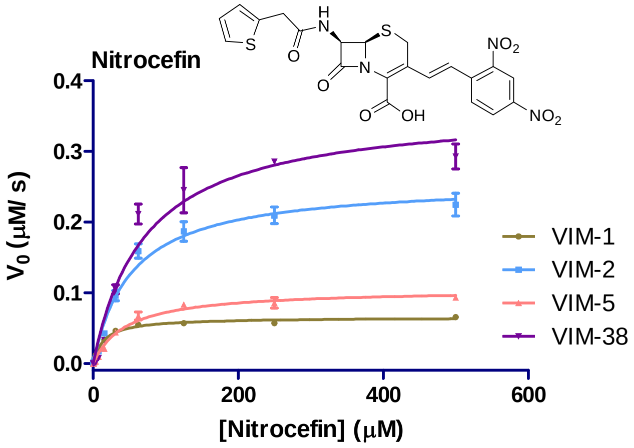

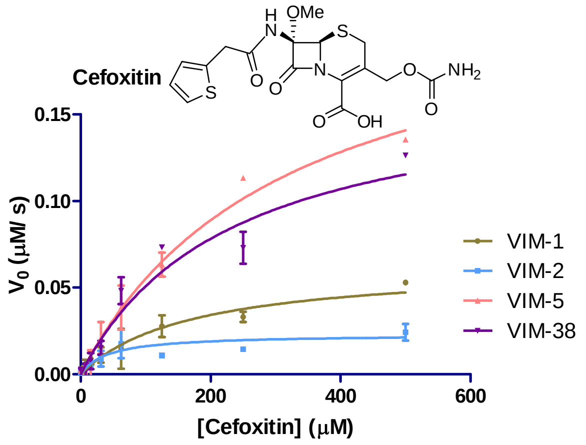

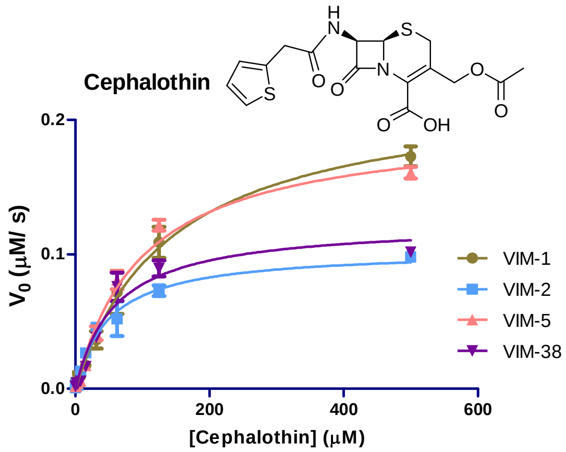

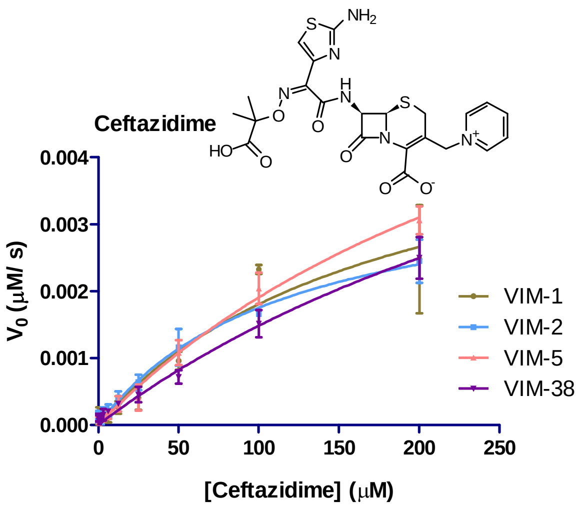

*Apparent kinetic parameters were reported for ampicillin and ceftazidime hydrolysis. #*C*-terminal His$_6$-tagged VIM-1 was used for kinetic analyses.

Table S5. Apparent K_D curve fitting for the binding of inhibitors (1) and (2) to VIM-2 and VIM-5.

<table>
<tr><td>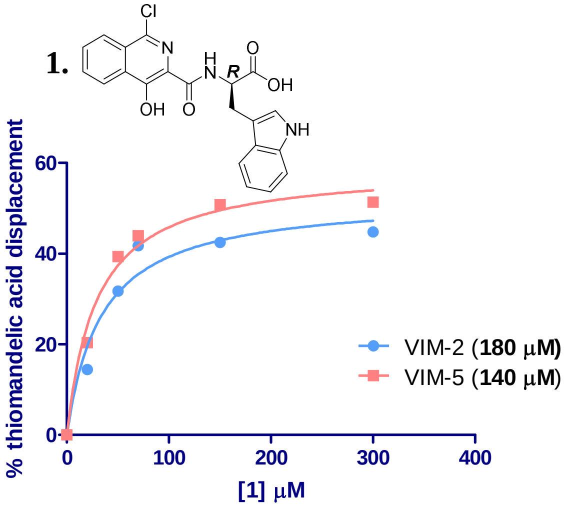
</td><td>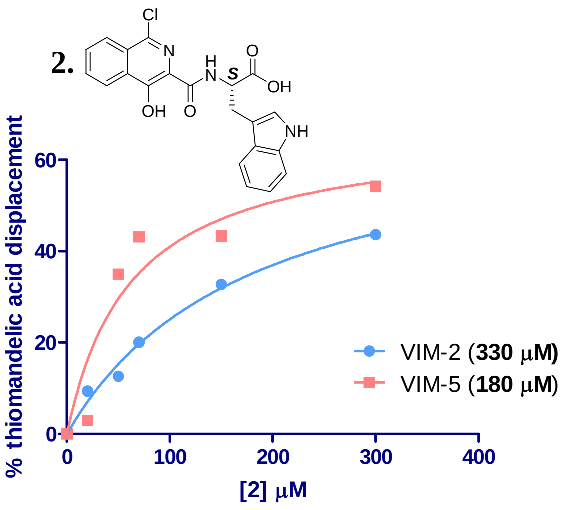
</td></tr>
<tr><td colspan="2">Apparent K_D fitting as monitored by ^{1}H CPMG NMR spectra of the signal recovery of thiomandelic acid (50 μM) upon addition of increasing concentrations of 1 and 2 with a final enzyme concentration of 50 μM VIM-2/VIM-5. Assays were conducted in 50 mM Tris-D_{11} (pH 7.5), and 0.02 % NaN_3 in 90 % H_2O and 10 % D_2O.</td></tr>
</table>

Table S6: Crystallography data collection and refinement statistics of PDB code: 5A87.

**Data set**	**VIM-5**
Data collection	
Resolution range (Å) (outer shell)	39.90-1.50 (1.54-1.50)
Space group	P 1 2_1 1
Unit cell dimensions	
a,b,c (Å)	41.27, 78.96, 64.03
α,β,γ (°)	90, 104.82, 90
No. of molecules/ASU	4
No. of unique reflections	61764
Completeness (%)	97.4 (96.6)
Redundancy	7.0 (7.1)
R_{merge}	0.132 (1.499)
Mean I/σ(I)	9.7 (1.2)
Wilson B value ($Å^2$)	14.86
**Refinement**	
R_{factor}	0.1552 (0.2655)
R_{free}	0.1915 (0.2949)
RMSD	
Bond length (Å)	0.008
Bond angle (°)	1.166
No. of atoms	7266
Protein	3487
Ligand	24
Ion	5
Water	408
$\langle B_{factor} \rangle$ ($Å^2$)	
Protein	19.03
Ligand	44.44
Ion	16.5
Water	33.77

Numbers in parentheses refer to the highest resolution shell.

R_{merge} is the unweighted R-value on I between merged reflections.

$R_{factor} = \Sigma hkl \mid |F_{obs} (hkl)| - k |F_{calc} (hkl)| \mid / \Sigma hkl |F_{obs} (hkl)|$ for the working set of reflections

R_{free} is the R-value for 3.8% of the reflections excluded from refinement.

Table S7: Comparison of kinetic parameters from this study (shaded rows) with data from previous studies (non-shaded rows).*

Substrate	K_m (µM)			k_{cat} (s^{-1})			k_{cat}/K_m ($\mu M^{-1} \cdot s^{-1}$)		
	**VIM-1**	**VIM-2**	**VIM-5**	**VIM-1**	**VIM-2**	**VIM-5**	**VIM-1**	**VIM-2**	**VIM-5**
Meropenem	130 ± 20	20 ± 4	70 ± 10	50	7	16	0.35	0.31	0.22
	48	5	49	13	1.4	2.4	0.27	0.28	0.05
Imipenem	6 ± 11	33 ± 4	58 ± 6	30	95	58	0.55	2.87	1
	1.5	10	12	2	9.9	3.5	1.3	0.99	0.29
Ampicillin	>1000	>500	>1000	122	180	230	0.1	0.25	0.2
	917	ND#	125	37	ND	14	0.04	ND	0.11
Ceftazidime	180 ± 60	120 ±20	340 ± 75	3	2	4	0.02	0.02	0.01
	794	98	149	60	89	0.2	0.076	0.9	0.001

*The previously reported kinetic data for VIM-1, VIM-2 and VIM-5 are from Franceschini et al. (4), Poirel et al. (5) and Garcar et al. (6), respectively. #ND, not determined.

REFERENCES

1. **Gouet P, Robert X, Courcelle E.** 2003. ESPript/ENDscript: Extracting and rendering sequence and 3D information from atomic structures of proteins. Nucleic Acids Res. **31**:3320-3323.

2. **Lassaux P, Hamel M, Gulea M, Delbruck H, Mercuri PS, Horsfall L, Dehareng D, Kupper M, Frere JM, Hoffmann K, Galleni M, Bebrone C.** 2010. Mercaptophosphonate compounds as broad-spectrum inhibitors of the metallo-β-lactamases. J. Med. Chem. **53:**4862-4876.

3. **Leiros HK, Edvardsen KS, Bjerga GE, Samuelsen O.** 2015. Structural and biochemical characterization of VIM-26 shows that Leu224 has implications for the substrate specificity of VIM metallo-β-lactamases. FEBS J. **282:**1031-1042.

4. **Franceschini N, Caravelli B, Docquier JD, Galleni M, Frere JM, Amicosante G, Rossolini GM.** 2000. Purification and biochemical characterization of the VIM-1 metallo-β-lactamase. Antimicrob. Agents Chemother. **44**:3003-3007.

5. **Poirel L, Naas T, Nicolas D, Collet L, Bellais S, Cavallo JD, Nordmann P.** 2000. Characterization of VIM-2, a carbapenem-hydrolyzing metallo-β-lactamase and its plasmid- and integron-borne gene from a *Pseudomonas aeruginosa* clinical isolate in France. Antimicrob. Agents Chemother. **44**:891-897.

6. **Gacar GG, Midilli K, Kolayli F, Ergen K, Gundes S, Hosoglu S, Karadenizli A, Vahaboglu H.** 2005. Genetic and enzymatic properties of metallo-β-lactamase VIM-5 from a clinical isolate of *Enterobacter cloacae*. Antimicrob. Agents Chemother. **49**:4400-4403.
